# Pulvinar neuromodulation for seizure monitoring and network modulation in temporal plus epilepsy

**DOI:** 10.1002/acn3.51815

**Published:** 2023-05-25

**Authors:** Yash Shashank Vakilna, Ganne Chaitanya, Muhammad Ubaid Hafeez, Adeel Ilyas, Manojkumar Saranathan, Jay Gavvala, Nitin Tandon, Sandipan Pati

**Affiliations:** ^1^ Texas Comprehensive Epilepsy Program, Department of Neurology The University of Texas Health Science Center at Houston Houston Texas USA; ^2^ Department of Neurosurgery, Heersink School of Medicine University of Alabama at Birmingham Birmingham Alabama USA; ^3^ Department of Radiology University of Massachusetts Chan Medical School Worcester Massachusetts USA; ^4^ Texas Comprehensive Epilepsy Program, Department of Neurosurgery The University of Texas Health Science Center at Houston Houston Texas USA

## Abstract

Deep brain stimulation (DBS) is a promising treatment for drug‐refractory epilepsies (DRE) when targeting the anterior nuclei of thalamus (ANT). However, targeting other thalamic nuclei, such as the pulvinar, shows therapeutic promise. Our pioneering case study presents the application of ambulatory seizure monitoring using spectral fingerprinting (12.15–17.15 Hz) recorded through Medtronic Percept DBS implanted bilaterally in the medial pulvinar thalami. This technology offers unprecedented opportunities for real‐time monitoring of seizure burden and thalamocortical network modulation for effective seizure reduction in patients with bilateral mesial temporal and temporal plus epilepsies that are not suitable for resection.

## Introduction

Deep brain stimulation (DBS) targeting the anterior nuclei of thalamus (ANT) has emerged as a promising treatment option for patients with drug‐refractory epilepsies (DRE).^1^ However, recent studies have shown that other thalamic nuclei, such as the pulvinar, also serve as potential neuromodulatory targets for seizure reduction based on their connectivity to the seizure network.^2^ The pulvinar nucleus is the largest thalamic nucleus and is anatomically connected to various cortical and subcortical regions.^3^, ^4^, ^5^, ^6^ Specifically, the pulvinar receives input from the visual, auditory, and somatosensory cortices. It has extensive connections with the parietal, temporal, and occipital cortices, particularly with the visual association areas. Among the subcortical structures, the superior colliculus, basal ganglia, pontine, and medullary reticular formation have asymmetric connections with the pulvinar. Finally, the pulvinar also has extensive connections with the contralateral pulvinar, allowing it to integrate information from both hemispheres. Given this extensive connectivity, the pulvinar is hypothesized to serve as a potential neuromodulatory target for patients with bilateral mesial temporal and temporal plus epilepsies that are not amenable to resection.^7^ In this study, we report a first‐in‐man application of ambulatory seizure monitoring using chronic sensing of local field potentials (LFPs) recorded from a Medtronic (Minneapolis, MN, USA) Percept DBS implanted in the pulvinar bilaterally. Initially, a spectral fingerprint (power in the band 12.15–17.15 Hz) was identified for reliable seizure detection in a patient with temporal plus epilepsy, confirmed after stereo‐electroencephalography (SEEG) evaluation. Temporal plus epilepsy is a type of drug‐resistant epilepsy that involves seizures originating in the mesial temporal lobe, with extension to adjacent brain regions(like insula, orbitofrontal or parietal regions). Subsequently, the patient underwent DBS implantation in bilateral pulvinar, and the fingerprints were deployed for seizure detection on the data recorded by the Medtronic Percept DBS. Spectral content in 12.15–17.15 Hz could detect all clinical seizures from pulvinar. Our findings demonstrate the feasibility of using chronic sensing of LFPs from the pulvinar for ambulatory seizure monitoring, providing unprecedented opportunities for monitoring seizure burden and modulating the thalamocortical network for seizure reduction.

## Case Study

**Figure 1:**
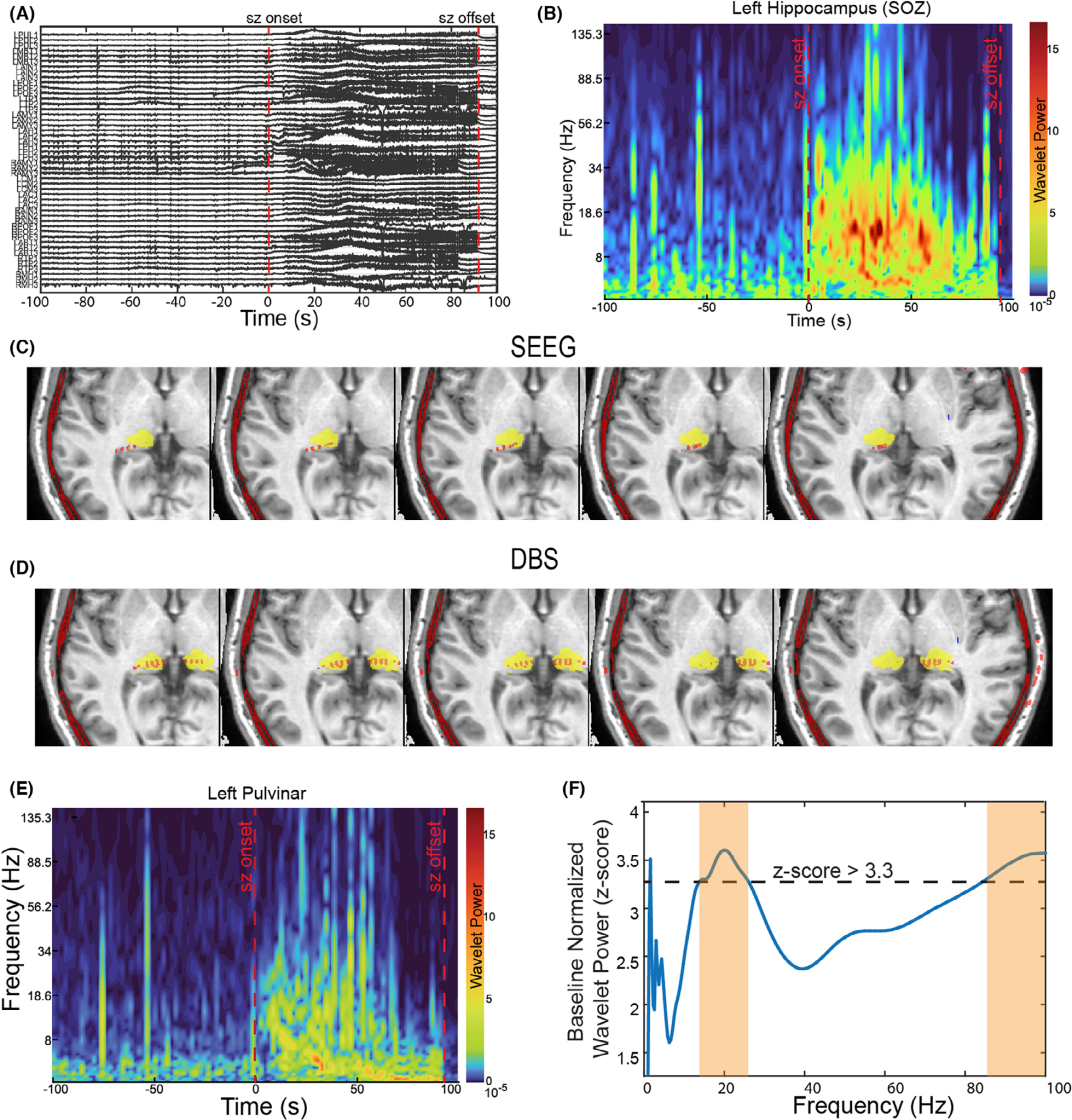
(A) The stereoelectroencephalography (SEEG) channel data demonstrating the onset, evolution, and termination of a clinical seizure. (B) Time–frequency spectrogram derived using Morlet wavelet to show the same seizure in the hippocampal SEEG channel. (C) Localization of the SEEG thalamic contacts in the left thalamic pulvinar. All four contacts were in the medial pulvinar, as confirmed by the THOMAS segmentation. (D) Localization of the Medtronic Percept deep brain stimulator (DBS) thalamic contacts in bilateral pulvinar. (E) Time–frequency spectrogram to show the same seizure in the pulvinar SEEG channel. (F) Baseline normalized wavelet power was estimated using the pulvinar SEEG data for the same seizure. At a threshold of 3.3 *z*‐score above the baseline, we noted a power increase in 2 distinct frequency bands: (1) 9.4–39.8 Hz and (2) 61.8–150 Hz. (Hz: Hertz, S: seconds, Sz: seizure).

A 28‐year‐old male patient with DRE since 18 years of age presented with episodes of focal impaired awareness seizures (FIAS) characterized by orofacial and bimanual automatisms, occurring once a week on average. His evaluation in the epilepsy monitoring unit (EMU) showed bilateral independent anterior temporal sharp waves and five habitual seizures that were either unlocalizable (*n* = 2) or had a left temporal (*n* = 2) or bitemporal onset. Magnetic resonance imaging showed evidence of atrophy and signal abnormality in the left anteromedial thalamus, as well as left mammillary body atrophy. Magnetoencephalography (MEG) showed bitemporal discharges, with a greater number of discharges on the left compared to the right. Positron emission tomography showed hypometabolism in bitemporal, left thalamic, and brainstem regions. He underwent SEEG leads placement with a hypothesis of possible left or bi‐mesial temporal lobe epilepsy (TLE). Given the possibility that this patient may require neuromodulation in the future and have an atrophied ANT, SEEG electrodes were implanted to sample the left pulvinar and centromedian nuclei. The SEEG confirmed seizure onset in the bilateral hippocampi and the right insula (TLE‐plus) epileptogenic network. Notably, the seizures from the left hippocampus recruited the left pulvinar nucleus (Fig. 1A,B,E). Given multifocal epilepsy, the patient was not considered a candidate for surgical resection and underwent placement of sensing DBS in bilateral pulvinar.

After 5 months following DBS implantation, he reported a 60% reduction in seizures, with the stimulation parameters set to a frequency of 145 Hz, pulse‐width of 90 μs pulse width, and current amplitude of 4 mA delivered with a stimulation cycle of 1 min ON and 4 min OFF.

## Methods

### Localization of pulvinar electrodes (SEEG and DBS)

Similar to our prior studies,^8^, ^9^ the post‐implant CT image was co‐registered to the pre‐implant structural MRI to confirm the localization of both the SEEG (Fig. 1C) and DBS electrodes (Fig. 1D) in the pulvinar. The thalamus‐optimized multi‐atlas segmentation (THOMAS)^10^ was used to create patient‐specific anatomically accurate pulvinar segmentation.

### Fingerprinting seizures from pulvinar SEEG

**Figure 2:**
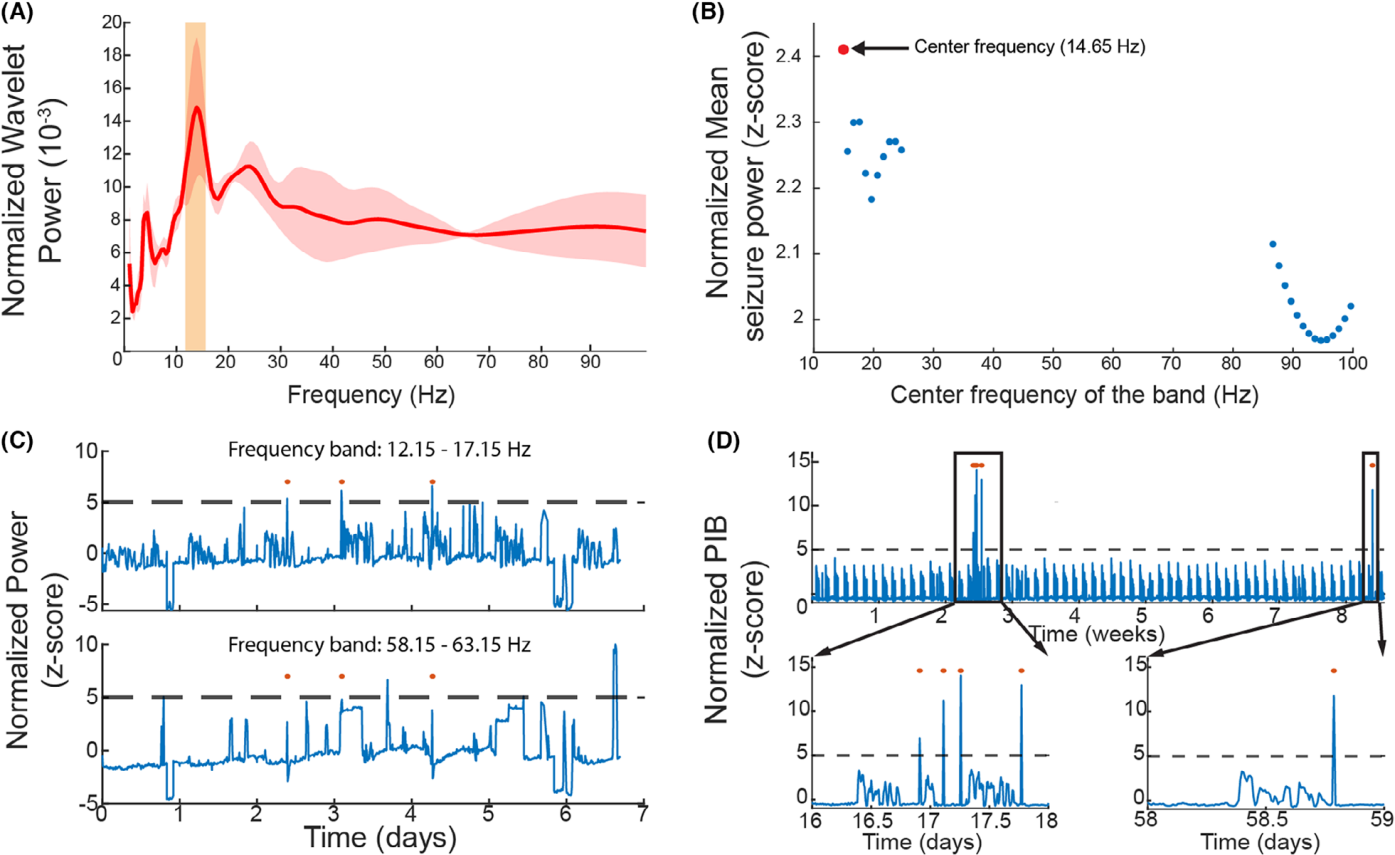
(A) Normalized power spectral density (PSD), computed using randomly selected windows across all three seizures, showing a peak in PSD at 12–17 Hz. (B) When the entire 7 days of SEEG data were analyzed using 5 Hz frequency bins, we noted that the average baseline normalized seizure power had a peak at central frequency of 14.65 Hz. This implies that the power in 5 Hz bands around 14.65 Hz, that is, 12.15–17.15 Hz, showed the maximum increase during the seizure when compared to interictal power. (C) Using the spectral fingerprint (12.15–17.15 Hz), all the 3 seizures were detected in the 7 days of SEEG data, using a *z*‐score threshold of greater than 5 times the baseline power. In contrast, no seizures were detected using a random frequency band (58.15–63.15 Hz). (D) The patient was sent home with his DBS sensing programmed to 12.15–17.15 Hz. During the 3‐month follow‐up, patient reported having 5 seizures, and the power in band 12.15–17.15 Hz, detected all the 5 seizures that were lateralized to the left pulvinar. There were no false positives.

To identify the spectral fingerprint that can be used for detecting seizures in the pulvinar (Fig. 1E), the wavelet power spectral density (PSD) of the SEEG time‐series was computed during the seizure (Morelet wavelet transform: central frequency: 1 Hz; full width at half maxima: 3 sec). This was *z*‐score normalized with respect to PSD of a 15‐min pre‐ictal baseline period. Significant frequency bands were identified using a threshold of >3.3 *z*‐score (>99 percentile increase in power) (Fig. 1F). In practice, Medtronic Percept DBS only allows sampling 5 Hz band power at any time. Hence, the statistically significant frequency bands were then subdivided into 5 Hz‐frequency bins, and a second‐level analysis was performed using wavelet PSD from all three seizures. The goal was to identify the 5 Hz‐frequency bin that best highlighted seizures. The band power time series was computed by bandpass filtering the signal in the 5‐Hz frequency bin using a finite impulse response filter and averaging the squared signal across non‐overlapping windows of 10 min across the 7 days of EMU admission (Fig. 2C).

### Programming the percept DBS for ambulatory sensing of seizures

Medtronic DBS device was configured to record the pulvinar power‐in band (PIB) time series in the statistically significant 5 Hz‐frequency bin (i.e., the spectral fingerprint of seizures in the pulvinar). The patient was monitored in the ambulatory setting with a fixed PIB sensing. The patient was asked to maintain a seizure diary to record all his seizures during this 3‐month interval. At the 3‐month follow‐up, the DBS data were downloaded, and the pulvinar PIB time series was correlated with the patient‐recorded seizures to determine the accuracy of detecting seizures in the pulvinar.

### Ethics

The Institutional Review Board approved the retrospective case study. The decision to implant the thalami during SEEG was based on clinical necessity, given the suspicion of multifocal epilepsy and atrophy of the anterior thalamus that would preclude the standard of care ANT DBS therapy.

## Results

### Spectral fingerprints of seizures recorded with pulvinar SEEG

Pulvinar‐SEEG data were used to estimate the normalized PSD frequency bands that distinguished seizures from the interictal state. At a threshold of 3.3 *z*‐score (>99 percentile increase) above the baseline, we noted a statistically significant power increase in 2 distinct frequency bands: (1) 9.4–39.8 Hz and (2) 61.8–150 Hz (Fig. 1F). Subsequently, when the power was computed in the 5 Hz‐frequency bins, PSD with a central frequency of 14.65 Hz (5 Hz bin: 12.15–17.15 Hz), showed the maximum increase during seizures compared to baseline (Fig. 2A,B). The 12.15–17.15 Hz band‐power filtered time series was isolated from the pulvinar‐SEEG. At a *z*‐score >5 (>99.99 percentile increase), it was able to delineate the accurate seizure time stamps in the 7 days of SEEG admission. Using this spectral signature, we were able to detect all SEEG‐recorded seizures (*N* = 3) (Fig. 2C).

### Chronic sensing of seizures from pulvinar DBS

When the patient was monitored for 3 months with pulvinar‐DBS configured to sense in 12.15–17.15 Hz frequency, we noted that the seizures reported in the seizure‐diary corroborated accurately with those detected by Percept DBS, using the *z*‐score threshold of >5. In the initial 3 months of the study, all reported seizures (*N* = 5) were found to be lateralized to the left pulvinar (Fig. 2D). However, as the study progressed, it was observed that seizures also occurred with lateralization to the right pulvinar (Fig. S1). There were no false positives detected (Fig. 2D).

## Discussion

The pulvinar, comprising 40% of the thalami, is the largest of the thalamic nuclei. The pulvinar is a heteromodal association nucleus with extensive reciprocal connectivity to the mesial and lateral temporal regions, cingulate cortex, inferior parietal, and dorsolateral prefrontal regions.^3^, ^6^, ^11^ The lateral pulvinar has connectivity to the visual cortex and inferior parietal regions.^12^ Pulvinar can modulate cortico‐cortical interactions and thalamocortical synchrony, and its recruitment during spontaneous limbic seizures has been confirmed with SEEG.^13^ Closed‐loop stimulation of the pulvinar and cortex has been shown to decrease posterior quadrant onset seizures.^2^ Stimulation of the medial pulvinar has demonstrated a reduction in the severity of seizures originating from the medial temporal lobe.^14^ In this study, we report the first‐in‐man application of ambulatory seizure monitoring using chronic sensing of LFPs from Medtronic Percept DBS implanted in the pulvinar bilaterally, characterizing the spectral fingerprints (power in band 12.15–17.15 Hz) for reliable seizure detection.

In our case, SEEG confirmed the presence of an epileptogenic network extending beyond the bi‐amygdala hippocampal regions to the right insula, precluding the selection of hippocampal RNS. Anterior thalamic atrophy, as confirmed by imaging, made ANT DBS unfeasible. Compared to the centromedian, the pulvinar demonstrated more significant recruitment of hippocampal seizures and interictals. This finding was the main reason for selecting the pulvinar over the centromedian as the target for neuromodulation. Our findings demonstrate the feasibility of pulvinar‐DBS for ambulatory seizure monitoring and modulation, providing a potential alternative to ANT for patients with drug‐refractory epilepsy, especially in those with bitemporal lobe epilepsy. This study also highlights the importance of thalamic sampling during SEEG in selected cases that can inform the selection of DBS targets.

The presence of an electronic seizure diary based on ambulatory electrocorticography from responsive neurostimulation (RNS) allows for monitoring of seizure burden, optimizing medications, differentiating seizures from coexisting nonepileptic spells,^15^ and lateralization of seizures for epilepsy surgery.^16^ However, chronic sensing from Medtronic Percept DBS (called BrainSense technology) should facilitate the development of an electronic seizure diary from an ambulatory thalamogram.^17^ After configuration in BrainSense Timeline mode, the neurostimulator measures raw time domain data sampled at 250 Hz, calculates the Fourier transform of the 10‐min signal, and stores only the relevant frequency band. Unlike the RNS device, where electrocorticogram (ECoG) data are directly visualized, the Percept device portrays ECoG data as spectral power within a specific 5 Hz band. A large‐scale study is required to validate the signatures from the thalamic pulvinar. The major limitations of this study are that it is a single case study and that the follow‐up period post‐DBS is relatively short, being less than 1 year.

In summary, we demonstrated the feasibility of chronic sensing of seizures from the pulvinar‐DBS in ambulatory settings. Pulvinar has extensive connectivity to various brain regions, and chronic sensing from Medtronic Percept DBS provides unprecedented opportunities for ambulatory seizure monitoring and modulation, providing a potential alternative to ANT for patients with drug‐refractory epilepsy.

## Author Contributions

YV, MS, and SP contributed to the conception and design of this study. YV and GC performed the analysis. SP, CG, NT, JG, and MUH coordinated the clinical assessment. SP, CG, YV, MUH, and AI drafted and initially revised the manuscript and figures. All authors contributed to case discussions and edited the manuscript.

## Funding Information

No funding information provided.

## Conflict of Interest

Nothing to report.

## Supporting information


Figure S1.
