# Supplementary material for: Pulvinar neuromodulation for seizure monitoring and network modulation in temporal plus epilepsy
Source: Ann Clin Transl Neurol. 2023 May 25;10(7):1254–9. doi: 10.1002/acn3.51815 (PMC10351657; doi:10.1002/acn3.51815)

Figure S1. Examples of seizures recorded from the right pulvinar deep brain stimulator. The red vertical line indicates patient-reported seizures that are represented by increased power in band in 12.15–17.15Hz


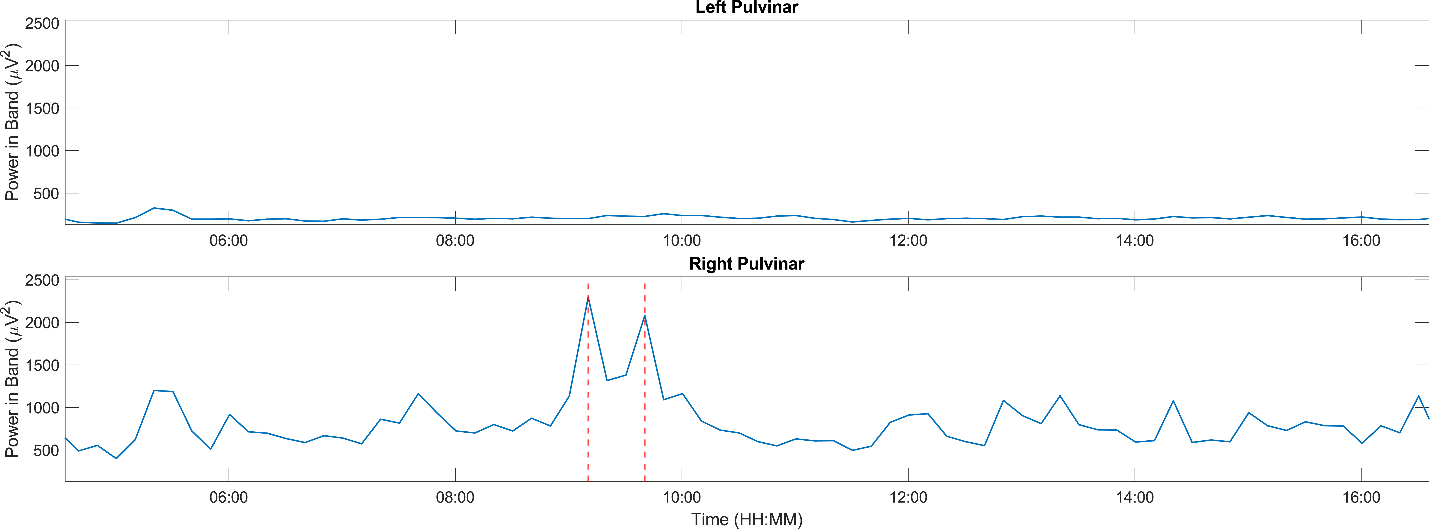


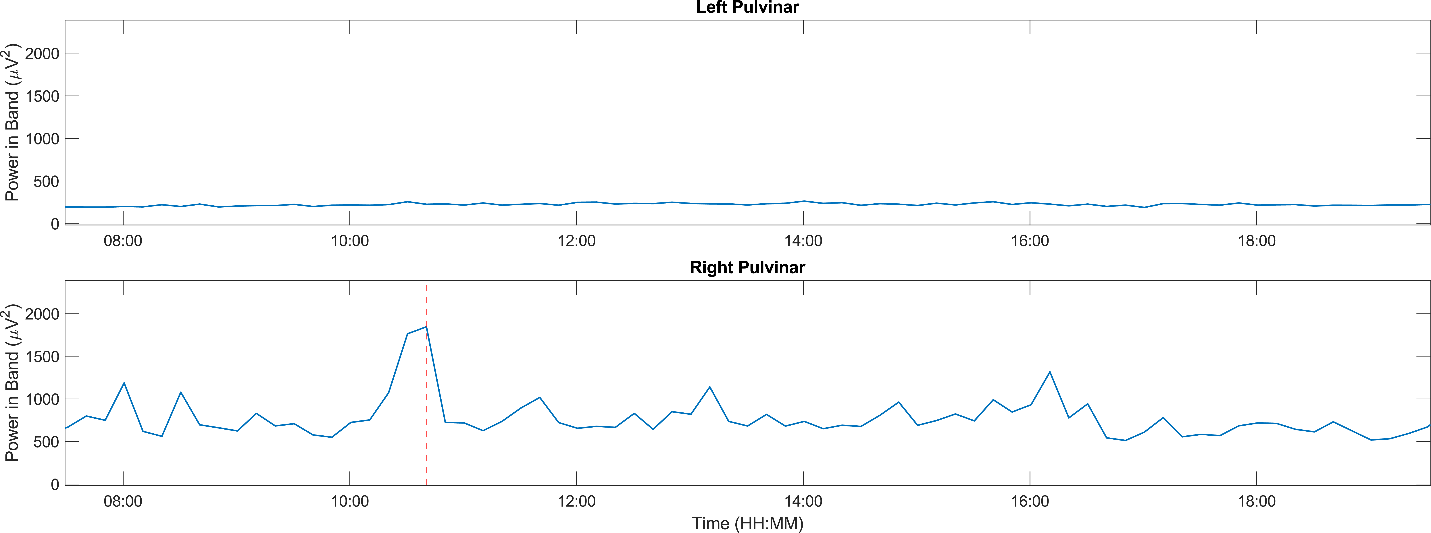

Supplement: Supplementary file 1 — Figure S1. [file ACN3-10-1254-s001.docx]
